# Moving Integrated Care for Pediatric Somatic Symptoms into Primary Care: An Innovative Approach

**DOI:** 10.1007/s10880-025-10115-y

**Published:** 2025-11-27

**Authors:** Thea Senger-Carpenter, Brittany Barber Garcia, Kira Sieplinga, Natoshia Cunningham

**Affiliations:** 1 College of Human Medicine, Department of Family Medicine, Michigan State University, East Lansing, USA; 2 Pediatric Behavioral Health, Helen DeVos Children’s Hospital, Grand Rapids, USA; 3 College of Human Medicine, Department of Pediatrics and Human Development, Michigan State University, East Lansing, USA; 4 Department of Pediatrics, Helen DeVos Children’s Hospital, Grand Rapids, USA

**Keywords:** Pediatric, Somatic symptom, Integrated care, Primary care, Behvioral, Psychological

## Abstract

Integrated behavioral healthcare has addressed common pediatric primary care concerns including anxiety and depression, but is infrequently applied to somatic symptoms (e.g., chronic pain, fatigue, or syncope unattributable to organic causes), which affect one in three youth. Developing an integrated care model for primary care–where most youth with somatic symptoms first present for evaluation and management–may increase access and positively impact child health. This manuscript summarizes the literature surrounding integrated care for pediatric somatic symptoms and proposes an adapted model for primary care. Drawing from the Pediatric Psychosocial Preventative Health model and cognitive-behavioral protocols for somatic symptom management, we propose that youth with mild to moderate symptoms can be effectively co-managed in primary care by a primary care provider delivering psychoeducation and facilitating team coordination, and an embedded behavioral healthcare provider conducting a brief, targeted intervention. This innovative approach leverages shared clinical responsibilities, as well as youth and families’ trust in the primary care setting, to accessibly deliver care for mild-moderate symptoms otherwise unaddressed in current management approaches. Implementation likely requires developing clinician support tools and identifying sustainable billing practices, but may result in accessible, holistic, care that curtails symptom persistence and/or progression.

## Introduction

Pediatric somatic symptoms are physical symptoms including fatigue, dizziness, abdominal pain, and syncope which are not fully explained by organic causes (Malas et al., 2017), and are frequently under-managed in typical approaches to care. Up to 31% of youth worldwide experience somatic symptoms, which account for 25 to 50% of primary care visits (Andresen et al., 2011; Rask et al., 2013; Vesterling et al., 2023). While severity varies, many youth experience significant functional impairment (e.g., school absenteeism, physical limitations) and psychosocial distress (e.g., anxiety/depression, social isolation) (Kangas et al., 2020; Moulin et al., 2015a). Somatic symptoms are also associated with increased healthcare utilization (Ibeziako & Bujoreanu, 2011). Indeed, youth with somatic symptoms use specialty and emergency care approximately twice as often as those without, incurring six to eight times the annual medical costs (Ahn et al., 2024; Saunders et al., 2020). Despite regular contact with medical care, many youth with somatic symptoms and their families feel insufficiently supported (Moulin et al., 2015b).

Promisingly, psychological approaches including cognitive-behavioral and dialectical-behavioral strategies may improve functioning and lower healthcare costs for youth with somatic symptoms (Fisher et al., 2018; Kitschen et al., 2024; Law et al., 2018). Yet, youth are typically referred to psychological services only *after* lengthy medical evaluations, which often entail extensive diagnostic tests/procedures and multiple evaluations with different providers, if they are referred at all (Malas et al., 2017). Such practices may convey that youth are being passed off to behavioral healthcare because no “real cause” has been identified, and that providers believe symptoms are imagined or intentional (Moulin et al., 2015b; Neville et al., 2019).

## Potential of the Primary Care Setting

Primary care may be the optimal setting in which to diagnose somatic symptoms and deliver psychological support. Because somatic symptoms are influenced by interacting biological, psychological, and social factors (Williams & Zahka, 2017), comprehensive approaches to diagnosis as well as management are critical (Sood et al., 2017; Voigt et al., 2010). Primary care providers are well-positioned to perform such comprehensive evaluations. Over 90% of United States children receive annual well visits, during which mental and physical health are routinely assessed (Foy et al., 2019; National Center for Health Statistics, 2025). This continuity facilitates the formation of trusted patient/provider relationships (Lines, 2022), and allows providers to consider physical symptoms within the broader context of psychological/behavioral and social factors and histories.

Interestingly, while most primary care providers regularly encounter somatic symptoms, few make a formal diagnosis (Malas et al., 2018), suggesting barriers such as low confidence in diagnostic/communication skills. However, a timely diagnosis may reduce youth and family distress, as well as the length of time youth go without effective symptom management. Further, establishing that somatic symptoms are a differential diagnosis, and that somatic and organic processes are not mutually exclusive, from the beginning sets the stage for appropriately limiting diagnostic procedures and introducing psychological strategies (Malas et al., 2017; Newlove et al., 2021; Ruth Saunders et al., 2025).

While many primary care providers refer patients with somatic symptoms for psychotherapy, the lack of providers trained in somatic symptom management and stigma associated with behavioral healthcare limit uptake (Kangas et al., 2020; Moulin et al., 2015b; Neville et al., 2019). Integrated care models, where medical and behavioral healthcare providers work together to support patients, may address some of these barriers (Lines, 2022; McLeigh et al., 2022). Most importantly, perhaps, integrated care models may reduce stigma associated with behavioral healthcare by relocating assessment and intervention into a trusted medical setting (Lines, 2022). Indeed, data indicate that most parents desire access to behavioral healthcare in primary care, even when their children do not exhibit clinically significant behavioral problems (Mehus et al., 2019). This may be especially critical in the context of somatic symptoms, as affected youth and families often feel stigmatized and neglected by medical providers, reducing their willingness to partner with providers in care (Moulin et al., 2015b). Integrating behavioral healthcare in to early phases of somatic symptom evaluation and management may help convey the importance of psychological approaches regardless of symptom etiology (Ruth Saunders et al., 2025). Indeed, integrated care models have the potential to synthesize behavioral and medical care—which are often conceptualized as related, but fundamentally separate, systems—into a more comprehensive approach to whole-person health. While integrated care may benefit a breadth of pediatric conditions given known impacts of social factors on symptoms and outcomes (Radtke et al., 2023; Rivera Rivera et al., 2024; Rubinstein et al., 2020), this shift may be particularly salient for somatic symptoms which are neither exclusively physical nor behavioral in origin and management.

Integrated care models have been applied in pediatric primary care to anxiety, depression, attention-deficit/hyperactivity disorder, and substance use (Hostutler et al., 2025; McLeigh et al., 2022; Yonek et al., 2020). Application to somatic symptoms remains uncommon, however, despite the fact that somatic symptoms may be equally or more common than these conditions (Ghandour et al., 2019; Lebrun-Harris et al., 2022; Vesterling et al., 2023). Though the degree of integration varies, medical and behavioral co-management featuring shared visits and documentation is considered the most effective for improving access and acceptability for pediatric patients and caregivers (Asarnow et al., 2015; Hostutler et al., 2025; McLeigh et al., 2022).

## Integrated Somatic Symptom Management

Among adults with somatic symptoms (e.g. pain, fatigue), a small number of pilot studies suggest the feasibility of integrating brief (i.e., three to five sessions) psychological interventions into primary care (Burton et al., 2012; Haun et al., 2024; Hubley et al., 2017). These included psychoeducation, collaborative goal setting, and cognitive-behavioral strategies delivered by embedded or remote mental health specialists, or a physician with specialized training. While these emerging data suggest positive impacts on patient functioning and symptom burden, it is essential to investigate an integrated primary care model specific to pediatric patients, given the importance of tailoring intervention to different stages of child development (Kangas et al., 2020).

Few studies have investigated integrated care for pediatric somatic symptoms. Weersing et al.,(2012) described the development of a six-session behavioral intervention for anxiety and functional abdominal pain in primary care, and its’ application to a single case study. The intervention, which included cognitive-behavioral and relaxation strategies, was delivered by a therapist with an unspecified degree of primary care provider involvement. While the patient experienced improvements in anxiety, depression, and somatic symptoms, it is impossible to derive conclusions regarding feasibility or efficacy. It is also noteworthy that the authors conceptualized somatic symptoms as part of an internalization spectrum (i.e., physiological manifestations of emotional processes), rather than as distinct biopsychosocial phenomena that may co-occur with, but are not inherently intrinsic to, anxiety or depression.

Only one randomized controlled trial has examined the delivery of cognitive-behavioral therapy for comorbid somatic symptoms and anxiety disorders in multiple medical settings that included primary care (Warner et al., 2011). The 10-week, 12-session Treatment of Anxiety and Physical Symptoms (TAPS) program, comprising relaxation training, cognitive restructuring, and exposure techniques was compared to a waitlist condition among 40 youth aged 8 to 16, recruited from gastroenterology, cardiology, and primary care clinics (Warner et al., 2011). While TAPS was acceptable and associated with decreased pain/discomfort and improved functioning, the length may impede delivery in primary care, which is often characterized by high volumes and a rapid pace. Instead, briefer, targeted interventions may be more impactful. Further, while participants could elect to receive TAPS in their medical clinic rather than a mental health office, only seven of the 20 participants did so, limiting the generalizability of these findings to primary care (Warner et al., 2011). Notably, TAPS addressed somatic symptoms and comorbid anxiety *disorders* (e.g., generalized anxiety disorder) (Warner et al., 2011). Interventions broadly addressing somatic and co-occurring *symptoms* may be more appropriate for primary care.

While integrated somatic symptom management remains understudied in primary care, evidence suggests such approaches positively impact youth in specialized outpatient (e.g., day programs, clinics) and inpatient settings (Kullgren et al., 2020a, 2020b; Weiss et al., 2024). For example, the adoption of a somatic symptom clinical pathway emphasizing medical/behavioral collaboration resulted in shorter stays and reduced median costs of $51,433 per inpatient and $6,075 per emergency department encounter in an academic children’s hospital (Kullgren et al., 2020a, 2020b). Additional support can be drawn from studies of youth with chronic pain (i.e., headaches, amplified musculoskeletal pain), which suggest that integrating pain-specific psychological approaches into pain clinics and treatment centers is associated with improved coping and functioning (Fisher et al., 2018; Weiss et al., 2024). These effects persist over time and are associated with in-person and remotely delivered care (Kashikar-Zuck et al., 2013; Law et al., 2017). Notably, Cunningham et al., (2021) recently found that a stepped-approach combining in-office psychoeducation and relaxation training with targeted cognitive-behavioral therapy was feasible and effective for youth with functional abdominal pain managed in gastroenterology, demonstrating the potential of integrating psychological approaches for chronic pain into outpatient medical settings.

Accumulating evidence supports the feasibility and benefit of integrating psychological approaches for pediatric somatic symptoms into inpatient and specialized outpatient settings. However, effective support should not be withheld until inpatient care is required, and specialized settings serve relatively few youth, belying the prevalence of somatic symptoms. Given these challenges, integrating psychological approaches for somatic symptoms into primary care, where most youth initially present for symptom evaluation and management (Kangas et al., 2020; Malas et al., 2017), may expand access and improve outcomes.

## Current Primary Care Practices

Despite the potential benefits, primary care-based approaches to somatic symptom assessment and management remain largely understudied or unadopted. While somatic symptoms are common and validated screening tools exist, there are no formalized recommendations for when or how to implement assessment in primary care. The limited available research recommends against lengthy medical evaluations in the absence of “red flags,” but provides little additional concrete guidance (Ibeziako & Bujoreanu, 2011; Malas et al., 2017). Instead, many providers pursue extensive diagnostic testing, driven by their own or families’ fear of missing an underlying organic cause (Malas et al., 2018; Neville et al., 2021). Repeatedly negative or inconclusive results often exacerbate youth and family anxieties, the lack of a diagnosis prompting many to seek specialist referrals (Ibeziako & Bujoreanu, 2011; Moulin et al., 2015b). As patients meet with additional medical providers who may offer different/conflicting explanations for somatic symptoms, they do not always feel confident in their care (Moulin et al., 2015b).

Regarding primary care management of pediatric somatic symptoms, the scant existing literature emphasizes close care coordination and strong therapeutic alliances, as well as limiting pharmaceutical intervention (Ibeziako & Bujoreanu, 2011; Malas et al., 2017, 2018). However, current medical research infrequently addresses how youth can manage symptoms to maximize functioning even as diagnostic processes unfold, despite the existence of effective psychological approaches. In the absence of concrete tools, youth may develop symptom-perpetuating management strategies (e.g., school avoidance, inactivity) (Vase et al., 2025). Thus, current assessment and management patterns may ensnare youth, families, and providers in a “vicious cycle” (Brownell et al., 2016) of mutual dissatisfaction, healthcare resource over-use, and functional impairment. An exception is the role that primary care providers play communicating with other providers and schools, particularly regarding strategies to support youths’ school attendance (Malas et al., 2017; Ruth Saunders et al., 2025). Importantly, data do not consistently support associations between school accommodations and attendance, due to variation in the types of accommodations provided and the potential for some common accommodations (i.e., sending symptomatic students home), to reinforce patterns of avoidance versus active coping (Logan et al., 2008; Vassilopoulos et al., 2021). Providers can, however, help youth, families, and school staff develop a plan for using coping skills during the school day (Li et al., 2021, 2024).

## A Proposed Model

**Fig. 1:**
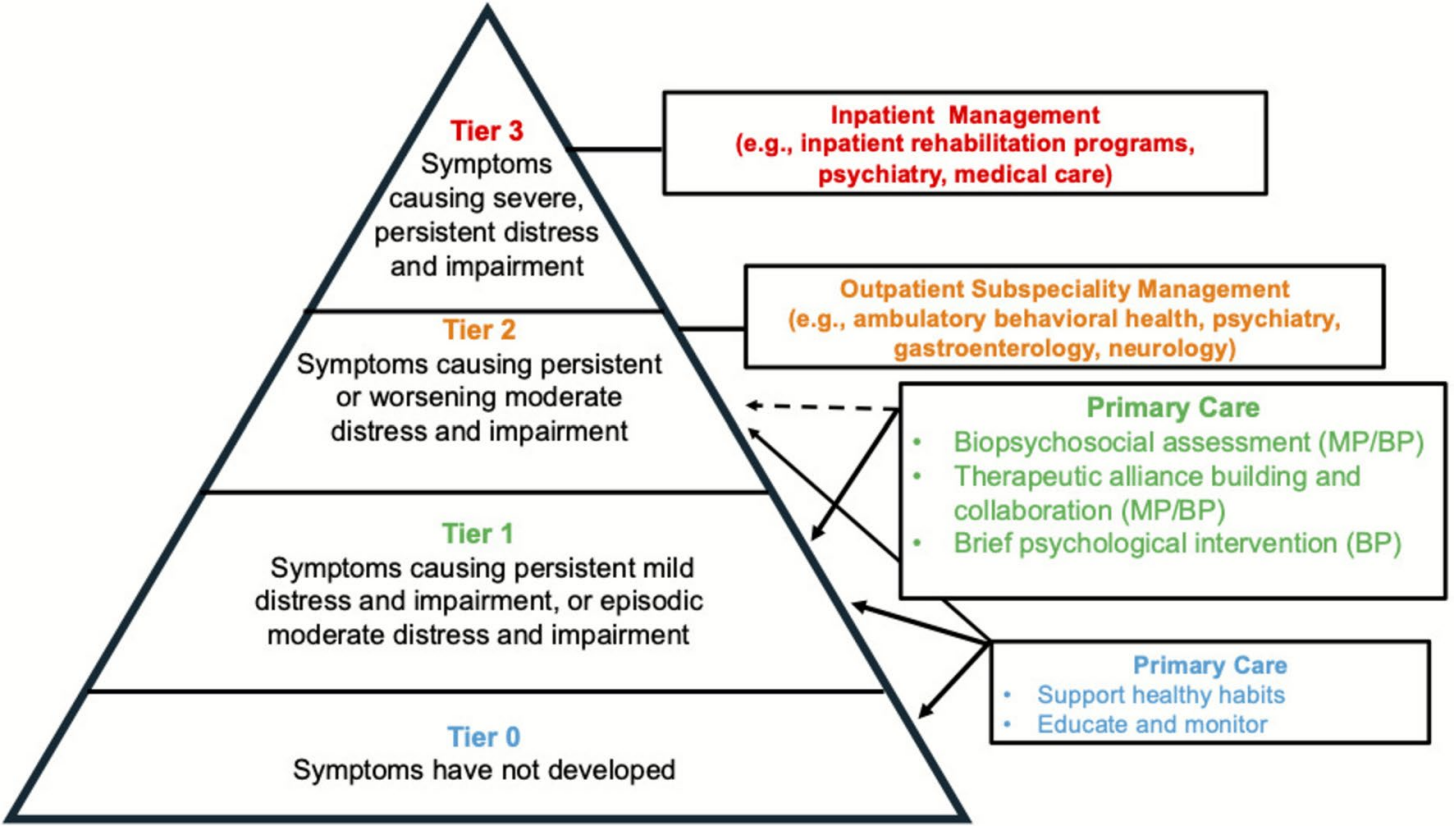
Conceptual model for integrated primary care management of pediatric somatic symptoms. *MP* medical providers, *BP* behavioral provider. Solid lines are areas of direct impact, while dotted lines represent the potential for primary care intervention to reduce the likelihood of escalation to Tier 2

**Fig. 2:**
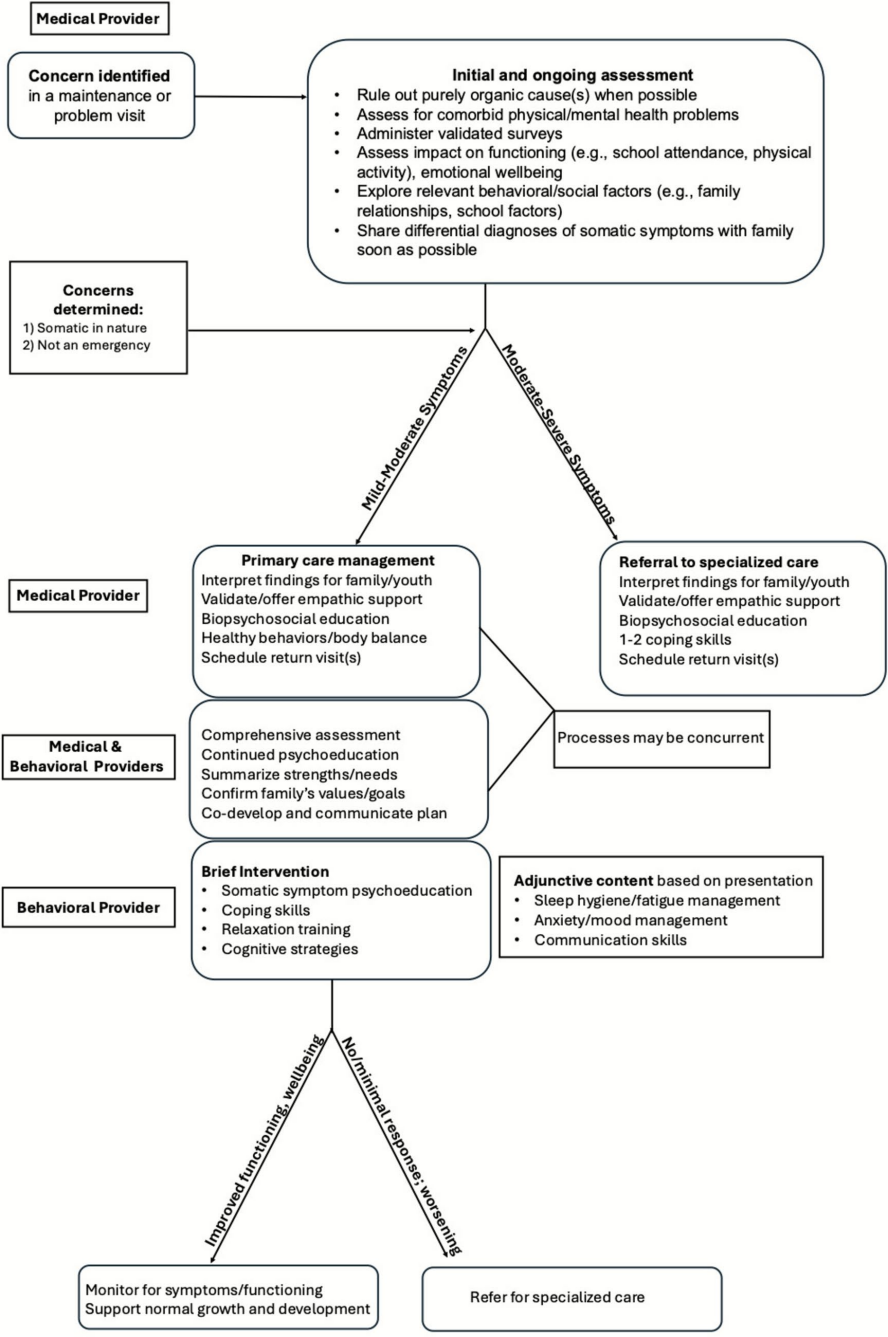
Proposed algorithm for integrated primary care somatic symptom management

We propose an innovative model of integrated care, where youth with somatic symptoms are managed by a primary care provider and an embedded behavioral healthcare provider (see Fig. 1). This model (see Fig. 2), guided by the Pediatric Psychosocial Preventative Health Model (Kazak et al., 2024) and the American Academy of Pediatrics’ (AAP) algorithm for integrating mental health care into practice (Foy et al., 2019), includes core components of measurement-informed, evidence-based care (Yonek et al., 2020), and draws from cognitive-behavioral protocols for somatic symptom management (Williams & Zahka, 2017).

A key adaptation from existing protocols is the distribution of content between a primary care and behavioral healthcare provider. All pediatric primary care patients may benefit from receiving education from their medical provider, while those with mild to moderate somatic symptoms would receive a brief, accessible intervention, potentially reducing their likelihood of requiring more specialized care. Finally, youth with more severe symptoms may receive education and a brief introduction to coping skills, bolstering their ability to manage distress while being referred for specialized support.

## Integrated Somatic Symptom Care Processes in Practice

### Assessment

Somatic symptom management would begin with the primary care provider’s initial assessment, triggered by a question or complaint during a health maintenance or problem-focused visit (see Fig. 2). Primary care providers are ideally positioned to conduct a comprehensive initial assessment given their familiarity with the child and family’s history. Indeed, assessing youths’ functioning, relationships, and psychological symptoms is already a pediatric primary care competency (Foy et al., 2019). Providers’ synthesis of findings across psychological, behavioral, and physiological domains may be supported by validated tools including the Children’s Somatic Symptom Inventory (CSSI) (Stone et al., 2019) or the Functional Disability Inventory (FDI) (Kashikar-Zuck et al., 2011). The CSSI probes the extent to which youth were bothered (0 = not at all, 4 = a whole lot) by 24 common somatic symptoms (e.g., dizziness, chest pain) in the past two weeks, while the 15-item FDI assesses difficulty (0 = no trouble, 4 = impossible) performing daily activities across home, school, recreational, and social settings over the “past few days” (Kashikar-Zuck et al., 2011; Stone et al., 2019). Both are suitable for ages 8–18 years, and have established clinical reference points for determining low, moderate, and high risk (Kashikar-Zuck et al., 2011; Stone et al., 2019). Importantly, the FDI has been integrated into routine care in pediatric gastroenterology, improving identification and management of at-risk patients (Cunningham et al., 2018). Information gathered via validated surveys, which can be distributed and completed ahead of time, may help providers make a “positive” diagnosis of somatic symptoms and determine whether youths’ presentations are appropriate for integrated primary care management or require referral to specialized care. Making this determination may also include assessing for comorbid mental and physical health concerns, leveraging existing screening processes in pediatric primary care (Foy et al., 2019).

## Psychoeducation

Following the initial assessment, primary care providers can share and contextualize their findings for families within the biopsychosocial model. The biopsychosocial model posits that somatic symptoms arise from, and are sustained by, interacting biological (autonomic nervous system dysregulation), psychological (co-occurring anxiety or depressive symptoms; coping patterns), and social (family dynamics, school relationships) factors (Williams & Zahka, 2017). When used as a framework for understanding and managing somatic symptoms, the biopsychosocial model addresses several gaps in current practices. Specifically, the biopsychosocial model encompasses symptom-sustaining or protective factors potentially missing from purely biomedical or psychosocial assessments, affirms the physical reality of symptoms, and may alleviate the ambiguity experienced by youth and families searching for a single explanatory cause (Førde et al., 2022). Achieving a shared biopsychosocial understanding of somatic symptoms may relieve diagnostic uncertainty such that patients and providers are less likely to pursue additional diagnostic testing or specialty referrals, since a positive diagnosis has been made and a clear treatment plan suggested (Neville et al., 2019, 2021). Establishing this foundational education, which can be reinforced by the primary care and behavioral healthcare provider throughout co-management, sets the stage for a treatment plan that addresses all three areas of the biopsychosocial model.

Critically, not all primary care or behavioral healthcare providers are familiar with, or confident in their ability to explain, the biopsychosocial model (Malas et al., 2018; Matthews et al., 2021). Targeted education focusing on the biopsychosocial model, efficacy/theoretical foundations of non-pharmacological somatic symptom management approaches, and strategies to communicate with patients and families has been shown to bolster provider confidence (Matthews et al., 2021). Such programming could be integrated into trainee curriculums and offered as continuing education but may have the greatest sustained impact if coupled with tools (e.g., handouts, conversational guides, electronic medical record templates) providers can use in practice.

## Care Coordination

The primary care provider can introduce integrated care as standard for pediatric somatic symptom management, as it directly addresses each biopsychosocial domain. The intervention itself can be framed as an opportunity to learn coping skills that may help youth manage symptoms and regain impaired functioning, while physical/mental health needs are still being addressed. This approach has been found acceptable by families of youth with chronic pain, a form of somatic symptoms (Claar & Scharff, 2007). As the intervention will be coordinated through primary care, primary care providers can offer reassurance of their ongoing involvement, reducing the likelihood that families feel passed off to a behavioral provider (Malas et al., 2017; Moulin et al., 2015b). When youth and/or families express reluctance or ambivalence, primary care providers can employ therapeutic communication and brief interventions (e.g., shared decision making, motivational interviewing) which are already important parts of practice (Foy et al., 2019).

## Promoting Healthy Habits

Drawing from the initial steps of evidence-based pediatric somatic symptom management protocols (Williams & Zahka, 2017), primary care providers or their delegate (e.g., registered nurses) can promote healthy habits including sleep hygiene, regular meals/hydration, and adequate physical activity integral to reestablishing body balance and a more positive mind–body connection.

## Intervention Content and Delivery

As a team (McLeigh et al., 2022), the primary and behavioral healthcare provider may share the initial intervention visit, completing a more thorough assessment and review of the biopsychosocial model of somatic symptoms. The behavioral healthcare provider would then conduct four to five one hour-long sessions delivering additional psychoeducation and core coping skills to all patients, adding adjunctive content (e.g., sleep hygiene, anxiety management) as needed. Opportunities for rapport building and empathic communication may be built into each session, as evidence suggests the provider/patient relationship is integral to somatic symptom management (Leaviss et al., 2020).

Proposed core skills, summarized in Table 1, are taken from established cognitive-behavioral and dialectical-behavioral approaches (Leaviss et al., 2020; Williams & Zahka, 2017), and support functioning across each biopsychosocial domain. Broadly, behavioral skills promoting relaxation (e.g., diaphragmatic breathing, progressive muscle relaxation) and activity pacing can help regulate physiological activation and limit symptom flares (Williams & Zahka, 2017). Cognitive skills including identifying/restructuring unhelpful thoughts, dialectical thinking (e.g. “these symptoms are hard AND I can cope”), and using grounding/relaxation skills to increase distress tolerance may target psychological symptoms-sustaining factors (Norman-Nott et al., 2025; Williams & Zahka, 2017). Finally, behavioral healthcare providers, youth, and families can work to support youths’ school attendance and social/extracurricular activity (Williams & Zahka, 2017). Given that many youth with somatic symptoms fall into patterns of activity avoidance (Malas et al., 2017; Williams & Zahka, 2017), staged re-engagement utilizing coping skills may be necessary. Similarly, behavioral healthcare providers may partner with caregivers to address other social factors (e.g., frequent caregiver “status checks” asking about symptoms; caregivers problem solving for youth) that may inadvertently perpetuate symptoms (Table 1).

**Table 1:** Integrated approach to somatic symptom (SS) management in pediatric primary care

Assessment and education				
**Setting**	**Provider(s)**	**Number of sessions**	**Aims**	**Content description**
Primary care office	Medical provider	1–2	Assessment Psychoeducation Promoting healthy habits	-Clinical interview and validated survey administration -Screen for comorbid conditions -Identify diagnosis of SS within the biopsychosocial model -Reinforce good body care (e.g., regular sleep, hydration, meals, and physical activity)
Primary care office	Medical provider & behavioral health provider	1	Psychoeducation Care coordination Support family relationships	-Continue to contextualize findings from the initial assessment within the biopsychosocial model -Identify interdisciplinary team approach and team roles -Values-based goal development -Introduce guidelines to help caregivers support youth
**Brief intervention**^**1**^				
**Setting**	**Provider(s)**	**Weekly session**	**Skills**	**Content description**
Primary care office or remote videoconferencing	Behavioral health provider	#1	Develop therapeutic alliance Psychoeducation Behavioral activation/redirection Relaxation skills I	-Develop rapport through empathy, sharing personal connection to the work, and review of joint goals -Briefly review the biopsychosocial model of SS -Introduce concepts of coping and distraction, with rationale -Diaphragmatic breathing
		#2	Relaxation skills II Activity pacing	*Youth may select skills that sounds most interesting:* -Guided imagery -5-4-3-2-1 and rainbow grounding -Progressive muscle relaxation -Link activity pacing with avoiding SS flares -Develop a schedule balancing rest and activity
		#3	Cognitive skills I	-Introduce cognitive-behavioral model -Identify automatic negative thoughts related to SS -Reframing with the Three “C’s”: Catching, Checking, Changing
		#4	Cognitive skills II	-Dialectical “and” statements -Problem identification/solution testing related to SS -Self-advocacy skills
Primary care office	Medical & behavioral health provider	#5	Review Maintenance planning	-Review/summarize youth’s understanding of SS and skills -Develop a plan to continue practicing skills -Anticipate and plan for future challenges -Follow-up measures to assess change

Approach assumes purely organic etiology has been ruled out when possible, and SS determination made by primary care provider

^1^Intervention sessions are once weekly, lasting ~ 1 h. Adjunctive content (e.g., sleep hygiene/fatigue management; behavioral and psychopharmacological support for comorbid anxiety or low mood) provided based on presentation

To maximize accessibility, skill-building sessions may be delivered in the primary care office or remotely, in line with emerging literature supporting the efficacy of hybrid approaches for pediatric behavioral healthcare (Miller et al., 2021). The behavioral and primary care provider would collaborate throughout, ideally ending with a shared visit focused on future planning, as per adult literature (Hubley et al., 2017). Youth would be monitored closely, and those with a lack of clinical response, new, or worsening symptoms would be referred for more intensive and specialized care (Yonek et al., 2020). Depending on the child’s presentation and response to intervention, the behavioral healthcare provider may also offer booster sessions, particularly around times when symptoms are expected to worsen (e.g., school transitions). 

## Next Steps

Critical next steps include testing this approach in real-world settings, utilizing the expertise of clinicians and people with lived experience. Incorporating behavioral, primary care provider, patient, and caregiver perspectives into intervention development, refinement, and testing may be essential to ensure appropriate content and distribution of responsibilities. Indeed, our team has developed a protocol for a brief, primary care-based intervention, which is being evaluated by focus groups of outpatient pediatric medical providers with iterative refinement and pilot testing to follow.

An additional consideration is the availability of embedded behavioral healthcare providers (Lines, 2022; Zhu et al., 2025). Only 35–37% of pediatric practices have on-site behavioral healthcare providers (Malas et al., 2018; Mayne et al., 2016). We propose that in this situation, care is provided remotely by an off-site provider, as per adult models (Haun et al., 2024). Alternatively, a member of the medical team (e.g., a physician, advanced practice provider) may receive training and supervision from a licensed behavioral healthcare provider (potentially remotely), building on their skills in patient communication, assessment, and brief office-based interventions (Foy et al., 2019). A similar approach was utilized in adult primary care (Burton et al., 2012), and has been suggested as a means of achieving the AAP Pediatric Mental Health Competencies (Green et al., 2019).

For some practices, taking steps to bolster primary care providers’ confidence formulating and communicating somatic symptom diagnoses may be more feasible, yet highly valuable. This may entail facilitating educational opportunities (e.g., in-office journal clubs, continuing education activities) and supplying providers with diagnostic and/or communication support tools, some of which may be embedded into electronic medical record systems. For example, Rome IV Criteria diagnostic ‘pocket cards’, conversation guides, and a phone application are available via the Rome Foundation (2021) to support providers managing disorders of gut-brain interaction (Koppen et al., 2017). Supporting providers thusly may reduce the time to a positive diagnosis, allowing providers to supply youth with education and management strategies (e.g., a relaxation skill; activity pacing strategies) as well as specialist referrals if necessary.

Future work may examine associations of implementing integrated care for somatic symptoms with changes in healthcare utilization and costs. It is possible that the timely delivery of effective psychological support may ameliorate symptoms or limit progression to the extent that patients are less likely use the emergency department or request diagnostic testing. Establishing these associations may be important for developing support for implementation of this approach across care systems. However, it may be critical to first secure the financial sustainability of integrated care models on a practice-level (Zhu et al., 2025). Financial barriers include up-front costs of hiring behavioral healthcare providers, increased administrative needs, and developing a nuanced understanding of billing codes (Lombardi et al., 2023; Zhu et al., 2025). The Collaborative Care Model and the billing codes used by embedded psychologists in an integrated primary care model may serve as blueprints (Hostutler et al., 2023; Lombardi et al., 2023), though policy-level changes in reimbursement rates may be necessary to realize wide-spread implementation (Zhu et al., 2025).

Challenges to the proposed model may include primary care providers’ heavy workload (Boudreau et al., 2022; Olfson, 2016). However, the development and integration of provider support tools may support more expedient diagnostic and communication processes, while the distribution of intervention content between a primary care and behavioral health provider may address workload concerns to some degree. Finally, it is critical to acknowledge that somatic symptoms presentations characterized by high levels of disability, acuity, and/or comorbidity are inappropriate for primary care management and may require specialized intervention (see Figs. 1 and 2).

## Conclusion

Integrating psychological approaches for pediatric somatic symptoms into primary care may increase the accessibility of evidence-based care for a common, often debilitating, condition. Improved accessibility may result in youth receiving care earlier, reducing the risk of symptom intensification and/or persistence to improve long-term health and functioning. Critically, integrated approaches may reduce stigma and increase understanding of a complex biopsychosocial phenomenon, resulting in better youth outcomes.

## Data Availability

No datasets were generated or analysed during the current study.
